# Assessing and Evaluating the Perfectionism Social Disconnection Model: Social Support, Loneliness, and Distress Among Undergraduate, Law, and Medical Students

**DOI:** 10.1177/07342829241244951

**Published:** 2024-04-07

**Authors:** Samantha Chen, Donald H. Saklofske, Gordon L. Flett, Paul L. Hewitt

**Affiliations:** 1 6221 University of Western Ontario, London, ON, Canada; 2 7991 York University, Toronto, ON, Canada; 3 175068 University of British Columbia, Vancouver, BC, Canada

**Keywords:** perfectionism, perfectionistic self-presentation, social support, loneliness, distress

## Abstract

The current research evaluates the Perfectionism Social Disconnection Model (PSDM) by considering the links between measures of trait perfectionism and perfectionistic self-presentation and measures of social support, loneliness, and distress in cross-sectional research. A particular focus is on perfectionism and levels of social support as assessed by the Social Provisions Scale. The current study also uniquely evaluates levels of perfectionism and perfectionistic self-presentation in undergraduate students, medical students, and law students. The results across samples provided evidence that loneliness mediates the link between interpersonal perfectionism and distress in keeping with the predictions of the PSDM. Correlational results found robust links between loneliness and low levels of social support. Moreover, socially prescribed perfectionism and perfectionistic self-presentation were associated negatively with social support, and this was especially evident in terms of the facet tapping the nondisclosure of imperfections. Group comparisons of perfectionism yielded few significant differences in accordance with expectations. Levels of perfectionism tended to be lower among medical students. However, the links between perfectionism and distress were clearly evident among undergraduates, medical students, and law students, thus attesting to the vulnerability of perfectionistic students in general. Overall, the results further confirm the relevance of perfectionism in distress among students and applicability of the PSDM in various types of students.

Research in the perfectionism field continues to grow exponentially and a large proportion of the studies that are conducted continue to indicate that certain elements of perfectionism are associated with vulnerability to distress among student populations. The established links with outcomes such as depression and suicide ideation continue to indicate that needing to be perfect and needing to seem perfect come with considerable emotional and motivational costs (see Casale et al., 2020; Flett et al., 2022; Hill et al., 2020). A related reason for concern about the well-being costs of perfectionism is that a widely cited meta-analysis of levels of trait perfectionism in college students indicates that there are temporal differences and that levels of trait perfectionism are substantially on the rise (see Curran & Hill, 2019).

Various models have been proposed to understand how and when perfectionism becomes associated with distress. One overarching goal is to identify processes and mechanisms that moderate or mediate the link between perfectionism and distress. The current work is designed to take a more detailed and refined approach that tests predictions derived from the Perfectionism Social Disconnection Model (PSDM).

The Perfectionism Social Disconnection Model (PSDM; Hewitt et al., 2006; Hewitt et al., 2017) is an integrative theoretical framework that describes how perfectionism generates psychological distress, dysfunction, and psychopathology. It is built on a conceptualization that distinguishes between elements of trait perfectionism (i.e., self-oriented, other-oriented, and socially prescribed perfectionism) and facets of perfectionistic self-presentation (i.e., promotion, nondisplay, and nondisclosure). The PSDM suggests that perfectionism is associated with interpersonal characteristics and behaviours that make it difficult for perfectionists with trait perfectionism and perfectionistic self-presentation to connect with others. Consequently, individuals with high levels of perfectionism may experience significant levels of social disconnection (i.e., feeling rejected, excluded, and unwanted by others), which then leads to the wide variety of maladaptive and negative outcomes that have been associated with perfectionism. Social disconnection can be objectively experienced (i.e., actual isolation) versus subjectively experienced (i.e., perceived isolation).

Several tests of the PSDM have been conducted and reported. Some studies have used social support as an indicator of social disconnection even though social support is not an explicit emphasis in accounts of the PSDM put forth by Hewitt and associates or a subsequent expanded version of the model (see Sherry et al., 2016). Measures of actual social disconnection or closely related constructs (i.e., loneliness) constitute more direct tests of the model. However, in the current work, we used the Social Provisions Scale (SPS; Cutrona & Russell, 1987) because it was created to reflect the social provisions that Weiss (1973) proposed as the processes and factors that protect people from experiencing social and emotional loneliness. Weiss (1974) outlined six social benefits that people can obtain through their social relationships with other people. Of course, being disconnected from others creates a distance not only from other people but also the positive things they can provide. Moreover, some social provisions in particular (e.g., social integration) seem especially relevant as proxy indicators of the presence or absence of social connection with others. Cutrona and Russell (1987) emphasized how having these social provisions can help people cope with and adapt to stress.

Some past research designed to evaluate the PSDM has considered perfectionism and social support. In a cross-sectional study of 222 undergraduates, Sherry et al. (2008) examined trait perfectionism as well as perceived support based on three SPS subscales (i.e., attachment, reliable alliance, and guidance) and received support assessed with the Inventory of Socially Supportive Behavior (Barrera et al., 1981). Socially prescribed perfectionism (i.e., the perception that others demand perfection from the self) was associated with lower levels of perceived support assessed by the three SPS subscales but not associated with received support. Moreover, perceived social support partially mediated the relationship between socially prescribed perfectionism and depressive symptoms.

More recently, Rnic et al. (2021) evaluated the PSDM in a longitudinal study with 447 community adults. At Time 1, the participants completed the Hewitt–Flett Multidimensional Perfectionism Scale (Hewitt & Flett, 1991), the Perfectionistic Self-Presentation Scale (Hewitt et al., 2003) and a measure of depressive symptoms. At Time 2, six months, participants completed measures of perfectionistic self-presentation, social disconnection indices, and depression. Social disconnection was assessed in terms of loneliness. The researchers also assessed social hopelessness, social assurance, and support provisions, but the only SPS subscale included was the reassurance of worth subscale (Cutrona & Russell, 1987). The overall results indicated that all perfectionism trait dimensions and perfectionism self-presentational styles were associated with greater depressive severity via one or more facets of social disconnection with social hopelessness and loneliness yielding the results most in keeping with the predictions of the PSDM. Parenthetically, the SPS reassurance of worth subscale was associated significantly with socially prescribed perfectionism and all three PSPS facets (r’s ranging from −.28 to −.40). Loneliness and reassurance of worth were also linked robustly (r = −.66), thus supporting the contention that low social provisions represent a form of social disconnection.

Although investigators have studied the PSDM for well over a decade, there are still many issues that remain to be tested. At present, the majority of PSDM studies have focused primarily on trait perfectionism (in particular, socially prescribed perfectionism), and do not generally incorporate the self-presentational facets of perfectionism despite theoretical and empirical evidence proposing that these components represent the interpersonal expression of perfectionism, and have important social consequences. The Rnic et al. (2021) is an exception because it measured trait and self-presentational perfectionism. Accordingly, the current research also includes trait perfectionism and perfectionistic self-presentation. In addition, participants completed all six subscales of the Social Provisions Scale in the present study, with the expectation that both trait perfectionism and perfectionistic self-presentation would be associated not only with loneliness but also lower scores across most if not all of the social provisions. Close examination of the SPS items indicates that it has many items that reflect the presence or absence of close caring connections with other people, especially in the negatively worded scale items (e.g., “There is no one who shares my interests or concerns,” “There is no one I can turn to for guidance in times of stress”).

Furthermore, the current research also evaluated the PSDM in terms of its applicability not only to undergraduate students but also to law students and medical students. Another related objective of this study was to examine group differences across law students, medical students, and undergraduate students. Historically, most perfectionism research with students has focused on undergraduate students, yet the pressures to be perfect may be greater and more salient for other students (e.g., graduate students, students in a professional program). It was hypothesized in the current study that law students and medical students may be particularly vulnerable to perfectionistic tendencies related to the competitive nature of their academic program, and thus, may report higher levels of perfectionism compared to the undergraduate sample. Specifically, interpersonal dimensions of perfectionism may be particularly relevant given the high standards and expectations of law and medical programs, therefore it was hypothesized that law and medical students may endorse greater levels of socially prescribed perfectionism, and perfectionistic self-presentational facets, particularly nondisclosure of imperfection and nondisplay of imperfection. While this hypothesis fits with accounts of the pressures facing medical students and law students in particular, anticipated differences have not always been found in research that considers medical students (e.g., Henning et al., 1998). Notably, a few studies have demonstrated that medical students may actually report lower levels of perfectionism compared to levels found in other groups. For example, Enns and colleagues (2001) reported that medical students showed lower Doubts about Actions as measured using the Frost MPS and lower evaluative concerns (a composite score using the socially prescribed perfectionism subscale from the Hewitt-Flett MPS, and subscales of Concern over Mistakes and Doubts about Actions from the Frost MPS) in comparison with general arts students. Seeliger and Harendza (2017) also found that a sample of 298 newly enrolled medical students reported significantly lower socially prescribed perfectionism than the general population. It has been suggested that the admissions process for medical school entry may select for adaptive academic functioning (grades and admission test scores) as well as adaptive interpersonal functioning (interviews and personal references), reducing the likelihood of individuals with high levels of maladaptive perfectionism being admitted (see Enns et al., 2001). Alternatively, success in the competitive process of applying to medical or law school programs may have a positive impact on students by reducing self-doubt and easing concerns about meeting others’ expectations of them. To our knowledge, no study to date has compared perfectionism scores of a law student sample with other populations.

In addition to evaluating levels of perfectionism, we anticipated that certain aspects of perfectionism would prove problematic for students of all types in terms of likely associations with loneliness and distress. That is, past findings linking trait and self-presentational perfectionism with distress should be generalizable and replicated in perfectionistic law students and medical students who need to be or seem perfect.

## Method

### Participants

Three samples were recruited. A sample of undergraduate students taking a psychology course was recruited at a large Ontario university. Participants were 667 undergraduate students (24.0% male, 75.3% female, 0.4% other). The average age was 18.42 years (*SD* = 1.44). The ethnicity of the sample was 52.8% Caucasian/European, 37.2% Asian, 1.8% Black/African, 1.8% Middle Eastern, 1.0% South Asian, 1.0% Latin American, .9% Aboriginal/First Nations, 1.5% Mixed, and 1.9% Other.

Another sample of university students were recruited from undergraduate medical programs and law programs in Ontario. The law sample was comprised of 180 students (25% male, 75% female), with an average age of 26.02 years (*SD* = 4.78). The ethnicity of the law sample was 60.7% Caucasian/European, 16.3% Asian, 6.2% Middle Eastern, 2.8% Black/African, 2.2% South Asian, 1.7% Latin American, 1.1% Southeast Asian, 1.1% Aboriginal/First Nations, 3.9% Mixed, and 4.0% Other. The distribution of class year was as follows: 37.2% in first year, 26.1% in second year, 32.8% in third year, 3.3% in fourth year, and 0.6% had completed the degree.

The medical sample was comprised of 154 students (22.1% male, 77.9% female), with an average age of 24.43 years (*SD* = 2.57). The ethnicity of the medical sample was 53.2% Caucasian/European, 31.2% Asian, 3.2% Middle Eastern, 1.3% Black/African, 3.9% South Asian, 0.6% Latin American, 0.6% Aboriginal/First Nations, 3.9% Mixed, and 1.9% Other. The distribution of class year was as follows: 29.9% in first year, 27.9% in second year, 26.0% in third year, 15.6% in fourth year, and 0.6% in fifth year.

### Measures

Participants were administered the following self-report questionnaires in a system-randomized order.

#### Trait Perfectionism

Trait perfectionism was measured using the Multidimensional Perfectionism Scale (Hewitt–Flett MPS; Hewitt & Flett, 1991). The Hewitt–Flett MPS has three 15-item subscales assessing self-oriented perfectionism (e.g., “When I am working on something, I cannot relax until it is perfect”), socially prescribed perfectionism (e.g., “I find it difficult to meet others’ expectations of me”), and other-oriented perfectionism (e.g., “Everything that others do must be of top-notch quality”). Responses were rated on a 7-point Likert-type scale ranging from 1 (*Strongly Disagree*) to 7 (*Strongly Agree*). The validity and reliability of the Hewitt–Flett MPS have been well-established in both clinical and non-clinical samples (Hewitt & Flett, 1991, 2004).

#### Perfectionistic Self-Presentation

Perfectionistic self-presentation was measured using the Perfectionistic Self-Presentation Scale (PSPS; Hewitt et al., 2003). The PSPS is a 27-item instrument comprised of three subscales measuring perfectionistic self-promotion (e.g., “I try always to present a picture of perfection”), nondisplay of imperfection (e.g., “I judge myself based on the mistakes I make in front of other people”), and nondisclosure of imperfection (e.g., “It is okay to admit mistakes to others”). Responses were rated on a 7-point Likert-type scale ranging from 1 (*Strongly Disagree*) to 7 (*Strongly Agree*). Evidence supports the validity and reliability of the PSPS (Hewitt et al., 2003).

#### Perceived Social Support

Perceived social support was assessed using the 24 item Social Provisions Scale (SPS; Cutrona & Russell, 1987) that measures the six social provisions outlined by Weiss (1973): attachment (emotional closeness from which one derives a sense of security; for example, “I feel a strong emotional bond with at least one other person”), social integration (a sense of belonging that stems from sharing similar interests, values or ideas; for example, “I feel part of a group of people who shares my attitudes and beliefs”), reassurance of worth (feeling important to or recognized by others due to one’s competence, skills, and value; for example, “I do not think other people respect my skills and abilities”), reliable alliance (assurance that others can be counted on in times of stress; for example, “There are people I can depend on to help me if I really need it”), guidance (receiving advice and/or information; for example, “There is someone I could talk to about important decisions in my life”), and opportunity for nurturance (the sense that others rely upon him/her for their well-being; for example, “There is no one who really relies on me for their well-being”). Responses were rated on a 4-point Likert-type scale ranging from 1 (*Strongly Disagree*) to 4 (*Strongly Agree*). The validity and reliability of the SPS have been well-established (Cutrona & Russell, 1987; Green et al., 2007; Vogel & Wei, 2005).

#### Received Social Support

Received social support was measured using the Inventory of Socially Supportive Behaviours (Barrera et al., 1981). The ISSB has 40 items that assess how often individuals received various forms of assistance during the preceding month (e.g., “Comforted you by showing you some physical affection”). Participants rate the frequency of each item on 5-point Likert-type scale ranging from 1 (*Not at all*) to 5 (*About every day*). Research supports the reliability and validity of the ISSB (Barrera et al., 1981; Finch et al., 1997).

#### Loneliness

Loneliness was measured using the 20-item revised UCLA Loneliness Scale (UCLA-R; Russell et al., 1980). It assesses subjective feelings of loneliness and feelings of social isolation. Responses were rated on a 4-point Likert-type scale ranging from 1 (*Never*) to 4 (*Often*). The scale has been demonstrated to have sufficient validity and reliability (Russell et al., 1980; Vassar & Crosby, 2008).

#### Depression

Depressive symptoms were measured using the 21-item Beck Depression Inventory (BDI-II; Beck, Steer, & Garbin, 1988). Participants rated each item on a 4-point scale ranging from 0 (an item reflecting no depressive symptoms; for example, “I do not feel sad”) to 3 (an item reflecting severe depressive symptoms; for example, “I am so sad and unhappy that I can’t stand it”). There is significant evidence supporting the reliability and validity of the BDI-II (e.g., Beck et al., 1988).

#### Psychological Distress

Psychological distress was measured using the 21-item short form of the Depression, Anxiety, and Stress Scale (DASS-21; Lovibond & Lovibond, 1995). This measure consists of three 7-item subscales assessing depression (e.g., “I felt down-hearted and blue”), anxiety (e.g., “I felt I was close to panic”), and stress (e.g., “I found it difficult to relax”). Participants responded to items using a 4-point Likert-type scale ranging from 0 (*Did not apply to me at all*) to 3 (*Applied to me very much, or most of the time*) to indicate the extent to which they experienced symptoms during the past month. Extensive research supports the reliability and validity of the DASS-21 in clinical and nonclinical samples (Antony et al., 1998; Lovibond & Lovibond, 1995).

### Procedure

Undergraduate students were recruited from the Psychology Research Participation Pool at Western University and directed to the online study. All students who had access to the research participation pool system were eligible to participate. Following the completion of the online study, participants were debriefed and compensated with one research credit for their participation.

Other participants were recruited from undergraduate medical programs and law programs in Ontario through mass email recruitment. Law students were attending the Juris Doctor (JD) degree programs at Western University, University of Toronto, Queen’s University, York University Osgoode Hall Law School, and University of Ottawa. Medical students were attending the Doctor of Medicine (MD) degree programs at Western University Schulich School of Medicine, University of Toronto, Queen’s University, and McMaster University Michael G. DeGroote School of Medicine. Participation was completely voluntary. Interested participants were directed to the online study. Following the completion of the online study, participants were offered the opportunity to place their name in a draw to win one of 25 gift cards (22 gift cards valued at $20 and three gift cards valued at $50).

## Results

### Data Screening

Initial data screening procedures included excluding participants who had completed the study in 10 minutes or under. In addition, participants with completion times that were over 15 hours were excluded. Participants whose progress was incomplete were also excluded.

Furthermore, based on suggestions by Meade and Craig (2012), participants identified as careless responders were subsequently removed from the sample. Specifically, these participants incorrectly responded to at least two of three attentional check items (e.g. “Respond ‘Strongly disagree’ to this item”), or responded “no” when asked at the end of the study whether their data should be used in analyses. Overall, out of pool of 810 general undergraduate students, 141 participants were removed for time-related issues, incomplete progress, or careless responding. From a total of 298 law students, 108 participants were removed for time-related issues, incomplete progress, or careless responding. From a total of 233 medical students, 79 participants were removed for time-related issues, incomplete progress, or careless responding.

### Preliminary Analyses

The means, standard deviations, alpha reliabilities, skew index, and kurtosis index for all major study variables are presented in Table 1 for general undergraduate students, law students, and medical students). Alpha reliabilities for all measures were adequate (with the majority being α ≥ .80) and were generally consistent with past research findings.

**Table 1.** Descriptive Statistics for Study Variables in General Undergraduate Students, Law Students, and Medical Students.

	Mean	SD	α	Skewness	Kurtosis
1. Self-oriented perfectionism	70.65	13.50	.89	−0.05	−0.16
2. Other-oriented perfectionism	58.95	9.60	.73	−0.26	1.02
3. Socially prescribed perfectionism	58.50	10.95	.81	0.04	0.33
4. Perfectionistic self promotion	42.80	10.60	.89	0.07	−0.09
5. Nondisplay of imperfection	46.30	10.10	.88	−0.18	0.29
6. Nondisclosure of imperfection	24.71	7.07	.80	0.38	0.56
7. Self-oriented perfectionism	73.50	17.25	.93	−0.32	−0.44
8. Other-oriented perfectionism	58.95	12.90	.84	0.18	−0.10
9. Socially prescribed perfectionism	59.10	14.70	.87	0.15	−0.30
10. Perfectionistic self promotion	43.90	12.50	.90	−0.14	−0.66
11. Nondisplay of imperfection	48.20	11.50	.89	−0.28	−0.54
12. Nondisclosure of imperfection	24.80	7.56	.80	0.40	0.50
13. Self-oriented perfectionism	68.55	13.35	.89	−0.16	−0.18
14. Other-oriented perfectionism	55.20	11.70	.83	0.11	−0.09
15. Socially prescribed perfectionism	54.30	11.55	.85	0.00	−0.04
16. Perfectionistic self promotion	40.10	9.60	.85	−0.19	−0.14
17. Nondisplay of imperfection	44.90	9.20	.85	−0.08	−0.40
18. Nondisclosure of imperfection	21.21	6.37	.79	0.31	0.65

*Note.* Means and standard deviations for general undergraduate students are in rows 1 to 6. Means and standard deviations for law students are in rows 7 to 12. Means and standard deviations for medical students are in rows 13 to 18.

### Group Differences Between Law, Medical, and Undergraduate Students

A series of one-way ANOVAs investigated possible group differences among the variables of interest across undergraduate, law, and medical students. For several of the analyses, the Levene’s test for equality of variances showed that the homogeneity of variances assumption was not met. As such, the Welch’s ANOVA was used. Tests of significance were also conducted using Bonferroni adjusted alpha levels of .0038 per test (using an alpha of .05 across 13 variables).

### Trait Perfectionism

The one-way ANOVA for self-oriented perfectionism revealed a significant main effect, *Welch’s F* (2, 299.39) = 4.52, *p* = .012, η^2^ = .010. This group difference became non-significant when using the Bonferroni adjusted alpha levels of .0038 per test. Significant group differences were also found for other-oriented perfectionism, *Welch’s F* (2, 285.53) = 8.08, *p* < .001, η^2^ = .016. Post hoc comparisons using the Games–Howell post hoc procedure showed that medical students reported lower levels of other-oriented perfectionism (*M* = 3.68, *SD* = .70) compared to both law students (*M* = 3.93, *SD* = .86) and undergraduate students (*M* = 3.93, *SD* = .64). Lastly, significant group differences were found for socially prescribed perfectionism, *Welch’s F* (2, 288.25) = 8.93, *p* < .001, η^2^ = .017. Games–Howell post hoc comparisons showed that medical students reported lower levels of socially prescribed perfectionism (*M* = 3.62, *SD* = .77) compared to both law students (*M* = 3.94, *SD* = .98) and undergraduate students (*M* = 3.90, *SD* = .73).

### Perfectionistic Self-Presentation

The one-way ANOVA for perfectionistic self-promotion revealed a significant main effect, *Welch’s F* (2, 307.20) = 6.21, *p* = .002, η^2^ = .011. Games-Howell post hoc comparisons showed that medical students reported lower levels of perfectionistic self-promotion (*M* = 4.01, *SD* = .96) compared to both law students (*M* = 4.39, *SD* = 1.25) and undergraduate students (*M* = 4.28, *SD* = 1.06). Significant group differences were also found for nondisplay of imperfection, *Welch’s F* (2, 308.75) = 4.14, *p* = .017, η^2^ = .009. This group difference became non-significant when using the Bonferroni adjusted alpha levels of .0038 per test. Lastly, significant group differences were found for nondisclosure of imperfection, *F* (2, 996) = 15.83, *p* < .001, η^2^ = .031. Post hoc comparisons showed that medical students reported lower levels of nondisclosure of imperfection (*M* = 3.03, *SD* = .91) compared to both law students (*M* = 3.44, *SD* = 1.08) and undergraduate students (*M* = 3.53, *SD* = 1.01).

### Social Disconnection

The one-way ANOVA of perceived social support revealed a significant main effect, *F* (2, 996) = 17.84, *p* < .001, η^2^ = .035. Post hoc comparisons showed that medical students reported greater levels of perceived social support (*M* = 3.45, *SD* = .40) compared to both law students (*M* = 3.32, *SD* = .47) and undergraduate students (*M* = 3.23, *SD* = .42). Significant group differences were also found for received social support, *Welch’s F* (2, 334.05) = 16.53, *p* < .001, η^2^ = .028. Games-Howell post hoc comparisons showed that undergraduate students reported higher levels of received social support (*M* = 2.64, *SD* = .73) compared to both law students (*M* = 2.34, *SD* = .62) and medical students (*M* = 2.47, *SD* = .63). Lastly, significant group differences were found for feelings of loneliness, *Welch’s F* (2, 299.31) = 4.77, *p* = .009, η^2^ = .010. This group difference became non-significant when using the Bonferroni adjusted alpha levels of .0038 per test.

### Psychological Distress

The one-way ANOVA of depressive symptoms as measured using the BDI-II revealed a significant main effect, *Welch’s F* (2, 356.84) = 28.21, *p* < .001, η^2^ = .033. Games–Howell post hoc comparisons showed that medical students reported lower levels of depressive symptoms (*M* = .40, *SD* = .33) compared to both law students (*M* = .61, *SD* = .45) and undergraduate students (*M* = .64, *SD* = .50). Significant group differences were also found for depressive symptoms as measured using the DASS-21, *Welch’s F* (2, 332.54) = 18.35, *p* < .001, η^2^ = .024. Games–Howell post hoc comparisons showed that medical students reported lower levels of depressive symptoms (*M* = 1.56, *SD* = .50) compared to both law students (*M* = 1.85, *SD* = .72) and undergraduate students (*M* = 1.85, *SD* = .68). In addition, significant group differences were found for symptoms of stress, *Welch’s F* (2, 329.83) = 28.09, *p* < .001, η^2^ = .040. Games–Howell post hoc comparisons showed that medical students reported lower levels of stress (*M* = 1.75, *SD* = .47) compared to both law students (*M* = 2.14, *SD* = .61) and undergraduate students (*M* = 2.06, *SD* = .61). Lastly, significant group differences were found for symptoms of anxiety, *Welch’s F* (2, 354.55) = 55.40, *p* < .001, η^2^ = .063. Games–Howell Post hoc comparisons showed that medical students reported lower levels of anxiety symptoms (*M* = 1.41, *SD* = .40) compared to both law students (*M* = 1.70, *SD* = .58) and undergraduate students (*M* = 1.83, *SD* = .61), and that undergraduate students reported greater levels of anxiety than law students.

### Correlational Analyses

Correlations among the variables are displayed in Table 2 for the undergraduate sample, Table 3 for the law sample, and Table 4 for the medical sample. In general, trait perfectionism (self-oriented perfectionism, socially prescribed perfectionism, and other-oriented perfectionism) and perfectionistic self-presentational facets (perfectionistic self-promotion, nondisclosure of imperfection, and nondisplay of imperfection) showed weak to strong positive relations with each other. Socially prescribed perfectionism, perfectionistic self-promotion, nondisclosure of imperfection, and nondisplay of imperfection were all negatively correlated with overall levels of perceived social support based on total social support scores. Self-oriented perfectionism and other-oriented perfectionism were not significantly associated with perceived social support. Received social support showed inconsistent associations with perfectionism dimensions across the student samples.

**Table 2.** Bivariate Correlations Among Study Variables in General Undergraduate Students.

	1	2	3	4	5	6	7	8	9	10	11	12	13	14
1. Self-oriented perfectionism	1.00													
2. Other-oriented perfectionism	.40^**^	1.00												
3. Socially prescribed perfectionism	.49^**^	.31^**^	1.00											
4. Perfectionistic self promotion	.56^**^	.25^**^	.49^**^	1.00										
5. Nondisplay of imperfection	.34^**^	.08^*^	.42^**^	.69^**^	1.00									
6. Nondisclosure of imperfection	.28^**^	.09^*^	.40^**^	.63^**^	.57^**^	1.00								
7. Perceived social support	.01	−.02	−.30^**^	−.19^**^	−.21^**^	−.42^**^	1.00							
8. Received social support	.05	.07	−.02	−.02	−.04	−.14^**^	.32^**^	1.00						
9. Loneliness	.08^*^	.01	.34^**^	.24^**^	.30^**^	.43^**^	−.72^**^	−.26^**^	1.00					
10. Depressive symptoms	.13^**^	.01	.35^**^	.29^**^	.37^**^	.33^**^	−.38^**^	−.05	.54^**^	1.00				
11. Stress	.26^**^	.09^*^	.37^**^	.30^**^	.37^**^	.27^**^	−.23^**^	.13^**^	.41^**^	.61^**^	1.00			
12. Depression	.14^**^	.01	.37^**^	.28^**^	.36^**^	.36^**^	−.43^**^	−.03	.60^**^	.77^**^	.73^**^	1.00		
13. Anxiety	.16^**^	.04	.34^**^	.30^**^	.35^**^	.32^**^	−.30^**^	.13^**^	.43^**^	.58^**^	.75^**^	.69^**^	1.00	
14. Psychological distress (DASS-21 total Score)	.20^**^	.05	.40^**^	.33^**^	.40^**^	.35^**^	−.36^**^	.08^*^	.54^**^	.73^**^	.91^**^	.90^**^	.90^**^	1.00

*Note.* **p < .001 level; *p < .05 level. *N* = 667.

**Table 3.** Bivariate Correlations Among Study Variables for Law Students.

	1	2	3	4	5	6	7	8	9	10	11	12	13	14
1. Self-oriented perfectionism	1.00													
2. Other-oriented perfectionism	.45^**^	1.00												
3. Socially prescribed perfectionism	.59^**^	.39^**^	1.00											
4. Perfectionistic self promotion	.67^**^	.38^**^	.61^**^	1.00										
5. Nondisplay of imperfection	.47^**^	.23^*^	.55^**^	.76^**^	1.00									
6. Nondisclosure of imperfection	.47^**^	.20^*^	.54^**^	.64^**^	.61^**^	1.00								
7. Perceived social support	−.02	.02	−.23^*^	−.16^*^	−.19^*^	−.35^**^	1.00							
8. Received social support	.19^*^	.16^*^	.10	.12	.00	−.10	.40^**^	1.00						
9. Loneliness	.12	.05	.36^**^	.22^*^	.32^**^	.36^**^	−.76^**^	−.30^**^	1.00					
10. Depressive symptoms (BDI-II)	.24^**^	.07	.44^**^	.33^**^	.42^**^	.34^**^	−.41^**^	−.08	.62^**^	1.00				
11. Stress	.29^**^	.23^*^	.41^**^	.34^**^	.38^**^	.26^**^	−.23^*^	.08	.40^**^	.66^**^	1.00			
12. Depression	.16^*^	.03	.37^**^	.25^**^	.35^**^	.26^**^	−.46^**^	−.16^*^	.66^**^	.81^**^	.62^**^	1.00		
13. Anxiety	.27^**^	.13	.39^**^	.36^**^	.40^**^	.30^**^	−.24^**^	.03	.38^**^	.63^**^	.75^**^	.61^**^	1.00	
14. Psychological distress (DASS-21 total Score)	.27^**^	.14	.44^**^	.36^**^	.43^**^	.31^**^	−.37^**^	−.03	.56^**^	.81^**^	.89^**^	.87^**^	.88^**^	1.00

*Note.* **p < .001 level; *p < .05 level. *N* = 180.

**Table 4.** Bivariate Correlations Among Study Variables for Medical Students.

	1	2	3	4	5	6	7	8	9	10	11	12	13	14
1. Self-oriented perfectionism	1.00													
2. Other-oriented perfectionism	.32^**^	1.00												
3. Socially prescribed perfectionism	.38^**^	.26^**^	1.00											
4. Perfectionistic self promotion	.53^**^	.33^**^	.36^**^	1.00										
5. Nondisplay of imperfection	.32^**^	.20^*^	.37^**^	.64^**^	1.00									
6. Nondisclosure of imperfection	.23^*^	.15	.34^**^	.60^**^	.48^**^	1.00								
7. Perceived social support	−.07	.05	−.21^*^	−.25^**^	−.21^*^	−.50^**^	1.00							
8. Received social support	−.17^*^	−.09	−.26^**^	−.25^*^	−.26^**^	−.39^**^	.46^**^	1.00						
9. Loneliness	.07	−.03	.16^*^	.32^**^	.32^**^	.47^**^	−.81^**^	−.44^**^	1.00					
10. Depressive symptoms (BDI-II)	.17^*^	−.09	.30^**^	.16^*^	.20^*^	.31^**^	−.47^**^	−.22^*^	.55^**^	1.00				
11. Stress	.12	.11	.23^*^	.04	.13	.06	−.20^*^	−.03	.28^**^	.55^**^	1.00			
12. Depression	.06	−.19^*^	.21^*^	.20^*^	.28^**^	.29^**^	−.56^**^	−.22^*^	.65^**^	.71^**^	.41^**^	1.00		
13. Anxiety	−.01	−.02	.11	.00	.15	.03	−.22^*^	.07	.25^**^	.44^**^	.57^**^	.44^**^	1.00	
14. Psychological distress (DASS-21 total Score)	.08	−.04	.24^*^	.10	.24^*^	.17^*^	−.42^**^	−.08	.50^**^	.72^**^	.82^**^	.79^**^	.81^**^	1.00

*Note.* **p < .001 level; *p < .05 level. *N* = 154.

One clearly distinguishing finding is that multiple measures of perfectionism were correlated significantly with elevated levels of stress among undergraduate students and law students, but these associations were not significant among medical students. Indeed, overall mean levels of perceived stress were lower among the medical students relative to the levels of stress for students in the other two groups, and, as noted about, the medical students reported greater perceived support. The differences evident here are important to keep in mind in terms of suggesting that the medical student sample may be quite distinct in terms of their characteristics and experiences at the time of participating in this study.

In general, socially prescribed perfectionism, perfectionistic self-promotion, nondisclosure of imperfection, and nondisplay of imperfection showed weak to moderate positive correlations with feelings of loneliness. Furthermore, socially prescribed perfectionism and all three facets of perfectionistic self-presentation tended to have moderate, positive associations with symptoms of depression as measured using the BDI-II and with the total DASS-21 score of psychological distress. In addition, both depression and psychological distress were negatively correlated with perceived social support, and positively associated with loneliness.

Additional correlational analyses were conducted to examine in more detail the various facets of perfectionism in terms of specific associations with scores on the SPS subscales. The results are shown in Table 5 for each of the three samples. The results follow the pattern outlined above in terms of links primarily being evident with socially prescribed perfectionism and the three PSPS facets. There is some variability but certain associations are consistent across samples. Regarding the social provision subscales, five of the six subscale measures had significant links with the interpersonal perfectionism dimensions. The nurturance subscale was the one subscale that was not statistically significant (i.e., having the opportunity to be nurturant with others). The most robust finding across the samples was the negative association between the nondisclosure of imperfections and the other five social provisions subscales. Other results not shown in Table 5 indicate that in each sample, loneliness was very strongly correlated with scores on the social provisions subscales. For instance, in the medical student sample, loneliness was correlated *r* = −.39 with the nurturance subscale, but the correlations between loneliness and the other five social provisions subscales ranged from *r* = −.58 to *r* = −.76.

**Table 5.** Bivariate Correlations Between Perfectionism and Social Provisions Scale Subscales.

	Perfectionism Measures					
SPS subscales	Self	Other	Social	Promote	NDisplay	NDisclose
Undergraduates						
Guidance	−.05	−.03	−.34**	−.21**	−.20**	−.44**
Reassurance	.06	−.06	−.27**	−.12	−.17**	−.30**
Social integration	.02	−.03	−.18**	−.14	−.16**	−.32**
Attachment	−.04	−.08	−.30**	−.24**	−.24**	−.41**
Nurturance	.08*	.08*	−.01	−.02	−.03	−.12
Reliable alliance	−.03	−.05	−.31**	−.16	−.17	−.36**
Law students						
Guidance	−.11	.04	−.16*	−.24**	−.13	−.47**
Reassurance	.00	.16	−.30**	−.20*	−.29**	−.40**
Social integration	−.05	.03	−.21*	−.23**	−.21**	−.43**
Attachment	−.04	.03	−.15	−.24*	−.41**	−.42**
Nurturance	−.01	−.01	−.03	−.06	−.09	−.19**
Reliable alliance	−.14	−.01	−.30**	−.27**	−.18*	−.49**
Medical students						
Guidance	−.04	−.01	−.22**	−.17*	−.18*	−.38**
Reassurance	−.09	.04	−.31**	−.19*	−.22**	−.26**
Social integration	−.11	−.04	−.32**	−.21**	−.21*	−.33**
Attachment	−.02	−.04	−.19*	−.11	−.18*	−.34**
Nurturance	.13	.10	−.10	.04	.00	.09
Reliable alliance	.01	.04	−.21**	−.13	−.13	−.28**

*Note.* *p < .05, **p < .01. The following abbreviations were used: Self (Self-Oriented Perfectionism), Other (Other-Oriented Perfectionism), Social (Socially Prescribed Perfectionism), Promote (Perfectionistic Self-Promotion), NDisplay (Nondisplay of Imperfections), and NDisclose (Nondisclosure of Imperfections).

### Mediation Analyses

Mediation analyses were conducted to examine the relationship between perfectionism traits (self-oriented perfectionism, socially prescribed perfectionism, and other-oriented perfectionism), perfectionistic self-presentation (perfectionistic self-promotion, nondisclosure of imperfection, and nondisplay of imperfection), indicators of social disconnection (perceived social support, received social support, and loneliness), and outcomes of depression and psychological distress. The hypothesis that social disconnection would mediate the relationship between perfectionism and outcomes of depression and psychological distress was assessed by examining the significance of indirect effects. Using bias-corrected bootstrapping, a total of 1000 replications were run in Mplus Version 7 (Muthén & Muthén, 2019). If the 95% bias-corrected bootstrapped confidence interval for an indirect effect does not contain zero within its upper and lower bounds, it suggests mediation.

### Depression in General Undergraduate Students

**Figure 1.**
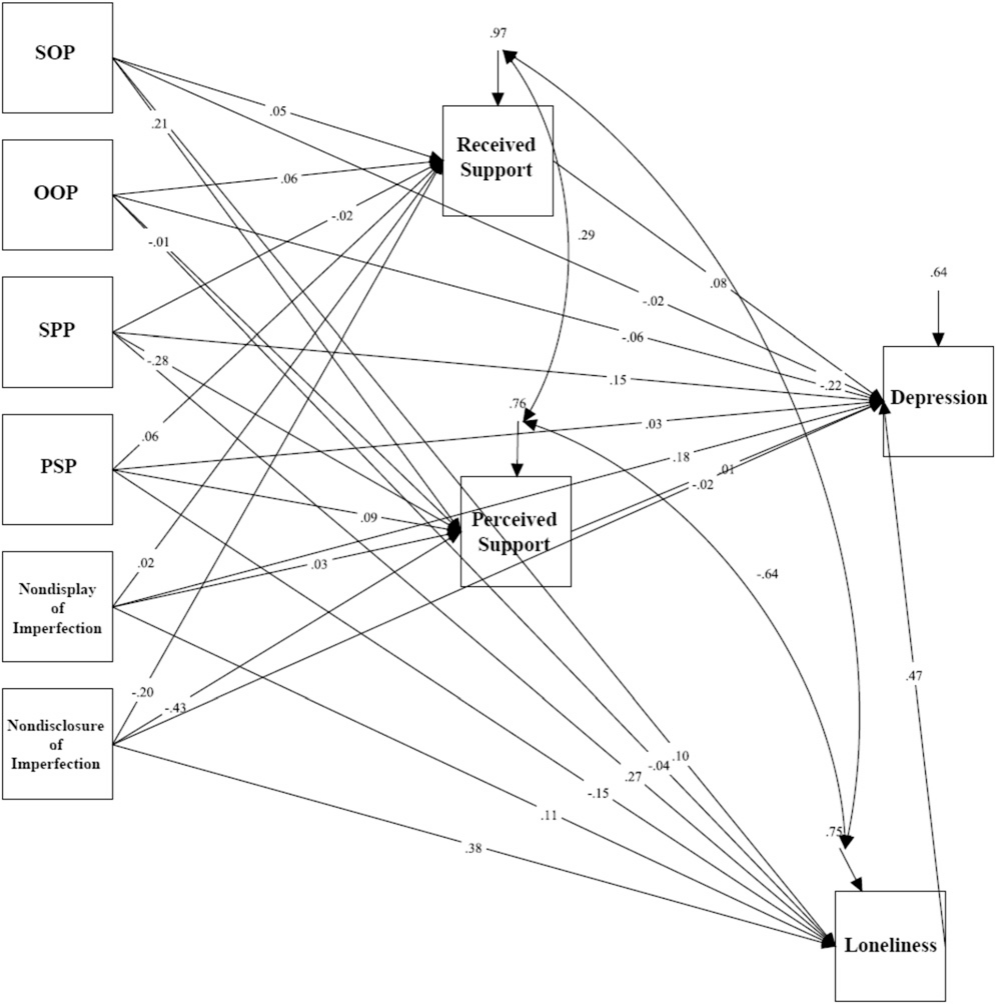
Mediation model examining trait perfectionism, perfectionistic self-presentation, social disconnection, and depression in general undergraduate students. Note. SOP = Self-Oriented Perfectionism; SPP = Socially Prescribed Perfectionism; OOP = Other-Oriented Perfectionism; PSP = Perfectionistic Self Promotion.

Mediation analyses tested a model examining the relationship between trait perfectionism, perfectionistic self-presentation, indicators of social disconnection, and depressive symptoms as measured using the BDI-II (see Figure 1). The model was just-identified (i.e., *df* = 0). Path analyses revealed a significant total effect between socially prescribed perfectionism and depressive symptoms as measured using the BDI-II via indicators of social disconnection, (*β* = .270, *p* < .001, 95% CI [.178, .361]). The standardized regression coefficient was significant for the specific indirect effect of loneliness: *β* = .128, *p* < .001, 95% CI [.078, .178]. The direct effect between socially prescribed perfectionism and depressive symptoms was significant, *β* = .146, *p* = .001, 95% CI [.059, .233]. These results indicate that the relationship between socially prescribed perfectionism and depressive symptoms was partially mediated by feelings of loneliness.

There was also a significant total effect between nondisplay of imperfection and depressive symptoms as measured using the BDI-II via indicators of social disconnection, (*β* = .236, *p* < .001, 95% CI [.139, .333]). The standardized regression coefficient was significant for the specific indirect effect of loneliness: *β* = .052, *p* = .037, 95% CI [.003, .101]. A significant direct effect between nondisplay of imperfection and depressive symptoms was also found, *β* = .181, *p* < .001, 95% CI [.090, .273]. These results indicate that the relationship between nondisplay of imperfection and depressive symptoms was partially mediated by feelings of loneliness.

Lastly, a significant total indirect effect between nondisclosure of imperfection and depressive symptoms via indicators of social disconnection, *β* = .139, *p* = .006, 95% CI [.040, .238] was found. The standardized regression coefficient was significant for the specific indirect effect of loneliness: *β* = .179, *p* < .001, 95% CI [.127, .230]. The direct effect between nondisclosure of imperfection and depressive symptoms was not significant, *β* = −.018, *p* = .700, 95% CI [–.112, .075]. These results indicate that the relationship between nondisclosure of imperfection and depressive symptoms was mediated by feelings of loneliness.

### Depression in Law Students

**Figure 2.**
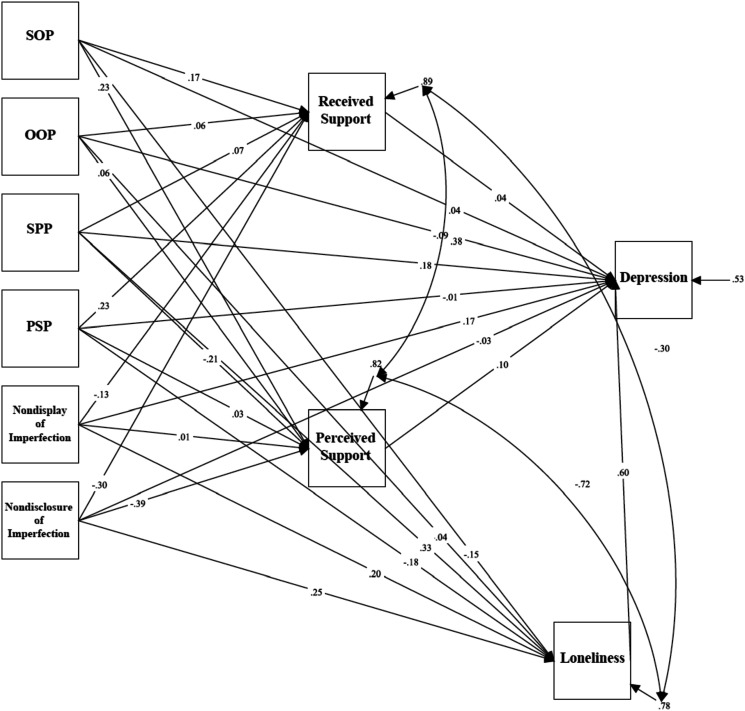
The mediation model examining trait perfectionism, perfectionistic self-presentation, social disconnection, and depression (BDI-II) for law students. Note. SOP = Self-Oriented Perfectionism; SPP = Socially Prescribed Perfectionism; OOP = Other-Oriented Perfectionism; PSP = Perfectionistic Self Promotion.

Mediation analyses tested a model examining the relationship between trait perfectionism, perfectionistic self-presentation, indicators of social disconnection, and depressive symptoms measured using the BDI-II in law students (see Figure 2). The model was just-identified (i.e., *df* = 0). Path analyses revealed a significant total indirect effect between socially prescribed perfectionism and depressive symptoms as measured using the BDI-II via indicators of social disconnection, *β* = 0.363, *p* < .001, 95% CI [.191, .536]. The standardized regression coefficient was significant for the specific indirect effect of loneliness: *β* = .195, *p* = .006, 95% CI [.056, .334]. The direct effect between socially prescribed perfectionism and depressive symptoms was significant, *β* = .185, *p* = .020, 95% CI [.030, .341]. These results indicate that the relationship between socially prescribed perfectionism and depressive symptoms was partially mediated by feelings of loneliness.

There was also a significant total effect between nondisclosure of imperfection and depressive symptoms via indicators of social disconnection, *β* = .072, *p* = .412, 95% CI [−.100, .244]. The standardized regression coefficient was significant for the specific indirect effect of loneliness: *β* = .152, *p* = .009, 95% CI [.037, .266]. The direct effect between nondisclosure of imperfection and depressive symptoms was not significant, *β* = −.030, *p* = .705, 95% CI [−.185, .125]. These results indicate that the relationship between nondisclosure of imperfection and depressive symptoms was mediated by feelings of loneliness.

### Depression in Medical Students

**Figure 3.**
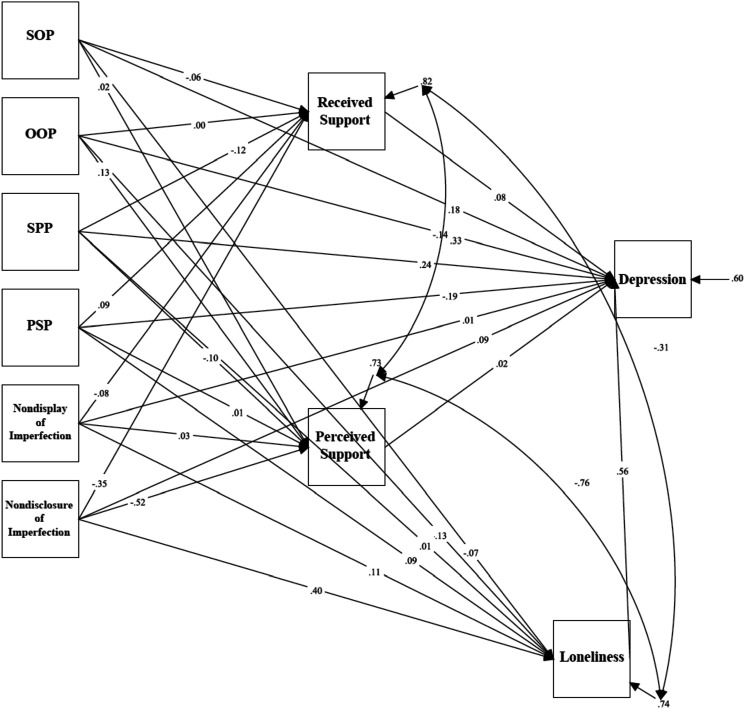
The mediation model examining trait perfectionism, perfectionistic self-presentation, social disconnection, and depression (BDI-II) for medical students. Note. SOP = Self-Oriented Perfectionism; SPP = Socially Prescribed Perfectionism; OOP = Other-Oriented Perfectionism; PSP = Perfectionistic Self Promotion.

Mediation analyses tested a model examining the relationship between trait perfectionism, perfectionistic self-presentation, indicators of social disconnection, and depressive symptoms measured using the BDI-II in medical students (see Figure 3). The model was just-identified (i.e., *df* = 0). Path analyses revealed a significant direct effect between self-oriented perfectionism and depressive symptoms (*β* = .178, *p* = .031, 95% CI [.017, .340]). In addition, there was a significant direct effect between socially prescribed perfectionism and depressive symptoms (*β* = .238, *p* = .004, 95% CI [.077, .399]).

There was also a significant total effect between nondisclosure of imperfection and depressive symptoms via indicators of social disconnection, *β* = .280, *p* = .004, 95% CI [.089, .470]. The standardized regression coefficient was significant for the specific indirect effect of loneliness: *β* = .223, *p* = .003, 95% CI [.074, .372]. The direct effect between nondisclosure of imperfection and depressive symptoms was not significant, *β* = .092, *p* = .364, 95% CI [-.106, .289]. These results indicate that the relationship between nondisclosure of imperfection and depressive symptoms was mediated by feelings of loneliness.

### Psychological Distress in General Undergraduate Students

**Figure 4.**
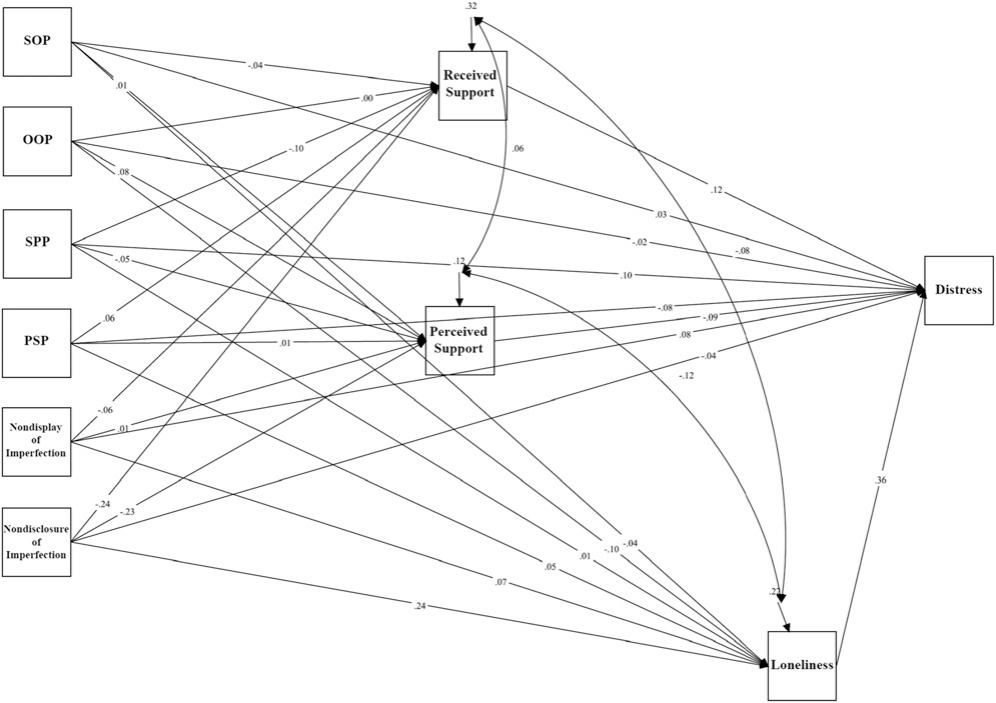
The mediation model examining trait perfectionism, perfectionistic self-presentation, social disconnection, and psychological distress for undergraduate students. Note. SOP = Self-Oriented Perfectionism; SPP = Socially Prescribed Perfectionism; OOP = Other-Oriented Perfectionism; PSP = Perfectionistic Self Promotion.

Mediation analyses tested a model examining the relationship between trait perfectionism, perfectionistic self-presentation, indicators of social disconnection, and psychological distress (see Figure 4). The model was just-identified (i.e., *df* = 0). Path analyses revealed a significant total effect between socially prescribed perfectionism and psychological distress as measured using the DASS-21 via indicators of social disconnection, (*β* = .286, *p* < .001, 95% CI [.190, .382]). The standardized regression coefficient was significant for the specific indirect effect of loneliness: *β* = .130, *p* < .001, 95% CI [.080, .181]. The direct effect between socially prescribed perfectionism and psychological distress was significant, *β* = .159, *p* < .001, 95% CI [.070, .249]. These results indicate that the relationship between socially prescribed perfectionism and psychological distress was partially mediated by feelings of loneliness.

There was also a significant total effect between nondisplay of imperfection and psychological distress via indicators of social disconnection, (*β* = .250, *p* < .001, 95% CI [.153, .347]). The standardized regression coefficient was significant for the specific indirect effect of loneliness: *β* = .053, *p* = .034, 95% CI [.004, .102]. A significant direct effect between nondisplay of imperfection and psychological distress was also found, *β* = .192, *p* < .001, 95% CI [.107, .276]. These results indicate that the relationship between nondisplay of imperfection and psychological distress was partially mediated by feelings of loneliness.

Lastly, a significant total indirect effect between nondisclosure of imperfection and psychological distress via indicators of social disconnection, *β* = .144, *p* = .005, 95% CI [.144, .243] was found. The standardized regression coefficient was significant for the specific indirect effect of loneliness: *β* = .182, *p* < .001, 95% CI [.132, .232]. The direct effect between psychological distress and nondisclosure of imperfection was not significant, *β* = .005, *p* = .905, 95% CI [–.083, .093]. These results indicate that the relationship between nondisclosure of imperfection and psychological distress was mediated by feelings of loneliness.

### Psychological Distress in Law Students

**Figure 5.**
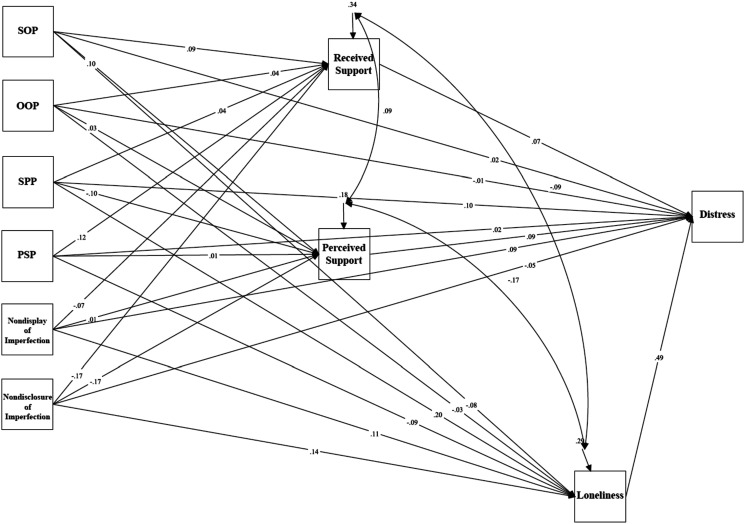
The mediation model examining trait perfectionism, perfectionistic self-presentation, social disconnection, and psychological distress for law students. Note. SOP = Self-Oriented Perfectionism; SPP = Socially Prescribed Perfectionism; OOP = Other-Oriented Perfectionism; PSP = Perfectionistic Self Promotion.

Mediation analyses tested a model examining the relationship between trait perfectionism, perfectionistic self-presentation, indicators of social disconnection, and psychological distress in law students (see Figure 5). The model was just-identified (i.e., *df* = 0). Path analyses revealed a significant total effect between socially prescribed perfectionism and psychological distress via indicators of social disconnection, *β* = .337, *p* < .001, 95% CI [.157, .517]. The standardized regression coefficient was significant for the specific indirect effect of loneliness: *β* = .178, *p* = .004, 95% CI [.056, .299]. The direct effect between socially prescribed perfectionism and psychological distress was significant, *β* = .170, *p* = .043, 95% CI [.005, .335]. These results indicate that the relationship between socially prescribed perfectionism and psychological distress was partially mediated by feelings of loneliness.

There was also a significant total effect between nondisclosure of imperfection and psychological distress via indicators of social disconnection, *β* = −.012, *p* = .902, 95% CI [-.201, .177]. The standardized regression coefficient was significant for the specific indirect effect of loneliness: *β* = .138, *p* = .009, 95% CI [.034, .241]. The direct effect between nondisclosure of imperfection and psychological distress was not significant, *β* = −.100, *p* = .266, 95% CI [-.276, .076]. These results indicate that the relationship between nondisclosure of imperfection and psychological distress was mediated by feelings of loneliness.

### Psychological Distress in Medical Students

**Figure 6.**
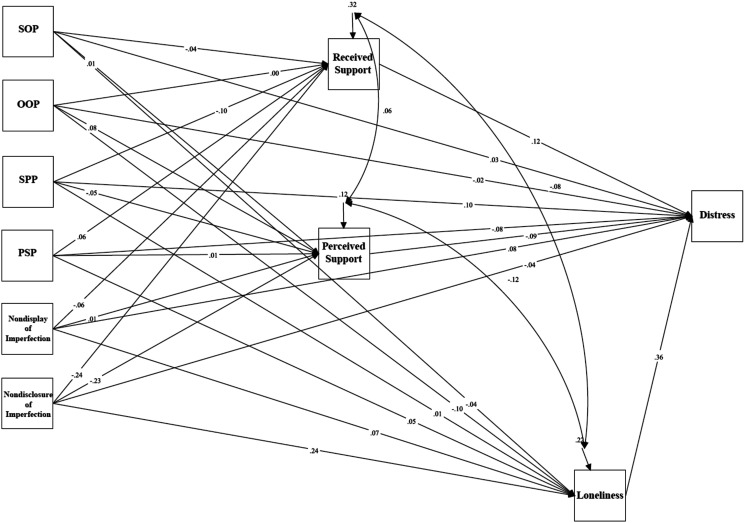
Study 3 mediation model examining trait perfectionism, perfectionistic self-presentation, social disconnection, and psychological distress for medical students. Note. SOP = Self-Oriented Perfectionism; SPP = Socially Prescribed Perfectionism; OOP = Other-Oriented Perfectionism; PSP = Perfectionistic Self Promotion.

Mediation analyses tested a model examining the relationship between trait perfectionism, perfectionistic self-presentation, indicators of social disconnection, and psychological distress in medical students (see Figure 6). The model was just-identified (i.e., *df* = 0). Path analyses revealed a significant direct effect between socially prescribed perfectionism and psychological distress, *β* = .204, *p* = .449, 95% CI [.017, .391].

There was also a significant total effect between nondisclosure of imperfection and psychological distress via indicators of social disconnection, *β* = .090, *p* = .401, 95% CI [−.120, .300]. The standardized regression coefficient was significant for the specific indirect effect of loneliness: *β* = .211, *p* = .008, 95% CI [.054, .368]. The direct effect between nondisclosure of imperfection and psychological distress was not significant, *β* = −.098, *p* = .368, 95% CI [–.311, .115]. These results indicate that the relationship between nondisclosure of imperfection and psychological distress was mediated by feelings of loneliness.

## Discussion

The current study examined the possible role of social disconnection as a mediator of the relationship between perfectionism and psychological outcomes undergraduate, law, and medical students. This study uniquely evaluated the Perfectionism Social Disconnection Model in terms of its generalizability and applicability not only to university students but also law students and medical students. A related objective of this work was to fully evaluate how trait perfectionism and perfectionistic self-presentation relate to the social provisions proposed by Weiss (1973).

Correlational analyses were conducted to examine associations among the variables of interest. Results for the three student samples showed that socially prescribed perfectionism and all three facets of perfectionistic self-presentation (perfectionistic self-promotion, nondisclosure of imperfection, and nondisplay of imperfection) were negatively correlated with perceived social support and positively correlated with loneliness. The level of received support was not linked with perfectionism, as was found in previous research by Sherry et al. (2008). This indicates that the perception or belief that others demand perfection as well as needing to seem perfect to others are associated with feeling disconnected to other people. Self-oriented perfectionism and other-oriented perfectionism were not significantly correlated with perceived social support. However, the associations between perfectionism and received social support were inconsistent across the student samples.

Strong links were found across the student groups between levels of loneliness and social support in terms of the SPS subscales. Loneliness and social support in terms of low social provisions were also associated with socially prescribed perfectionism and perfectionistic self-presentation. A closer examination of the various social provisions subscales found that nurturance scores were unrelated to perfectionism. However, across participant types, scores on four or five social provisions subscales were linked with socially prescribed perfectionism and perfectionistic self-presentation. This suggests that the social disconnection in interpersonal perfectionism extends to negative feelings and appraisals of social integration, guidance, reassurance, attachment, and reliable alliance. These are psychosocial themes to potentially explore when counselors and clinicians are probing the unmet interpersonal needs of distressed perfectionists.

Mediation analyses testing a model examining the relationship between trait perfectionism, perfectionistic self-presentation, indicators of social disconnection, and depressive symptoms showed that nondisclosure of imperfection was associated with depressive symptoms, and that feelings of loneliness mediated the links between perfectionism and depression across all three samples of law, medical, and general undergraduate students. Similar mediational results were also found for psychological distress, indicating that high levels of nondisclosure of imperfection are associated with a greater sense of social isolation that potentially increases the risk of experiencing psychological distress.

Findings from mediation analyses also suggest that law students show similar associations between socially prescribed perfectionism and depressive symptoms, as well as socially prescribed perfectionism and psychological distress as general undergraduate students. Specifically, results showed that beliefs that others have excessively high standards for oneself may lead to feelings of loneliness, which in turn confers vulnerability to depression and psychological distress in undergraduate and law students. Feelings of loneliness were not a significant mediator in the relationship between socially prescribed perfectionism and psychological distress, or in the relationship between socially prescribed and depression in the sample of medical students. In general, perceived social support and received social support were not significant mediators in the link between interpersonal perfectionism and psychological distress.

Collectively, the findings of the present study serve to expand the PSDM literature and to advance understanding of the interpersonal mechanisms through which perfectionism confers vulnerability for maladaptive psychological outcomes. Results indicate that feelings of loneliness are an important indicator of social disconnection and mediate the relationship between interpersonal dimensions of perfectionism and psychological distress. This is consistent with research showing that people high in perfectionism experience a powerful sense of not belonging and not being accepted by others (see Hewitt, 2020). Perfectionism may involve or promote an imbalanced behavioral pattern in which individuals turn inward and lead a life with a narrow focus on pursuing unrealistic goals and ruminating over perceived imperfections (Sherry et al., 2007). In such cases, striving for expectations may be prioritized over relationships, thereby increasing experiences of isolation and decreasing opportunities for connection. However, without positive connections to others, the present findings suggest that people high in interpersonal dimensions of perfectionism experience depressive symptoms and psychological distress.

### Group Comparisons of Undergraduates, Law Students, and Medical Students

One objective of this study was to examine possible group differences across the student samples. It was hypothesized that law students and medical students may be particularly vulnerable to perfectionistic tendencies related to the competitive nature of their academic program, and thus, may report higher levels of perfectionism compared to the undergraduate sample.

Tests of group differences across the three samples of law, medical, and general undergraduate students showed that medical students reported lower levels of other-oriented perfectionism, socially prescribed perfectionism, perfectionistic self-promotion, and nondisclosure of imperfection compared to both law and undergraduate students. These findings were contrary to study hypotheses that law students and medical students may report greater levels of socially prescribed perfectionism and perfectionistic self-presentational facets compared to general undergraduate students. At one level, these results illustrate the need to evaluate hypotheses than often go untested yet still go mostly unquestioned.

The anticipated group differences were not found, but the current study did yield considerable evidence that attests to why there should be substantial concern about the well-being of not only perfectionistic undergraduate students but also medical students and law students under pressure to be perfect and to present an image of perfection. It was found that medical students high in socially prescribed perfectionism and facets of perfectionistic self-presentation tend to have elevated levels of stress, distress, and loneliness. Similarly, law students high in socially prescribed perfectionism and facets of perfectionistic self-presentation tend to have elevated levels of stress, distress, and loneliness. It was also found uniquely among the law students in this study that self-oriented perfectionism was associated with higher levels of stress and depression. Given the links that were found between loneliness and lower reported received support and their links with facets of perfectionism, it is not too difficult to imagine a plethora of students (undergraduate students but also law students and medical students) who are perfectionistic, isolated, and avoidant. The present findings involving perfectionistic self-presentation, loneliness, and distress in law students and medical students are particularly unique.

It is also important to underscore the strong links found between trait perfectionism (self-oriented and socially prescribed perfectionism) and levels of perfectionistic self-perfectionism documented across groups. The tendency for students who must be perfect to also be invested in seeming perfect in terms of their self-presentations has several important implications. For instance, it is likely that when they are working in a professional capacity, perfectionistic self-presentation will come into play in terms of unwillingness among medical students and law students to admit to making mistakes. The sense of being under pressure to never be seen to be making a mistake and to being someone who is flawed and not perfect can be overwhelming for some students.

A related primary concern involves the likelihood that students high in perfectionistic self-presentation will be unable and unwilling to ask for help whenever it is needed. Law students and medical students who are perfectionistic and who are experiencing considerable psychological pain may be particularly likely to hide behind a front as their pressures mount and expectations become even more salient. It is easy to envision a high proliferation of students consumed with self-doubt in an environment of high perfectionistic self-presentation who mistakenly believe that other students are doing exceptionally well and are better able to handle extant pressures. All in all, while perfectionism levels were not found to be higher among law students and medical students, the pressures to be perfect and to seem perfect are highly problematic in terms of likely mental health costs, both now and in the future.

### Limitations

The current study has several limitations. For example, the generalizability and replicability of these findings needs to be evaluated, especially given indications that stress was not as evident among the medical students in this sample. Also, given that a cross-sectional design was used, the temporal relationships between perfectionism, social disconnection, and psychological distress were not established. Although the mediational sequence supported in the present studies were informed by theory and evidence, longitudinal designs are needed to test directional effects to see if the model replicates when predictors, mediators, and outcome variables are assessed at separate time points.

In addition, although the studies utilized well-established, reliable, and valid measures, they were primarily self-report instruments which may be biased towards impression management. Future research should incorporate methods of data collection that go beyond self-reports (e.g., informant reports).

### Conclusion

Overall, these results show that perfectionism facets involving interpersonal aspects related to the perceptions of others are particularly important to the PSDM, such as perceiving others demand perfection of oneself or managing one’s verbal disclosures to conceal one’s shortcomings or flaws. In particular, individuals who endorse high levels of these interpersonal dimensions of perfectionism report feeling interpersonally isolated, lonely, and alienated. The present study provided a greater understanding of the mechanisms underlying the PSDM by demonstrating how students in different types of academic programs who feel the pressure inherent in trying to be and appear perfect may be vulnerable to depression and psychological distress.
